# Myocardium at risk by magnetic resonance imaging: head-to-head comparison of T2-weighted imaging and early gadolinium enhanced steady state free precession

**DOI:** 10.1186/1532-429X-14-S1-O114

**Published:** 2012-02-01

**Authors:** Joey F Ubachs, Peder Sorensson, Henrik Engblom, Marcus Carlsson, Stefan Jovinge, John Pernow, Hakan Arheden

**Affiliations:** 1 Department of Clinical Physiology, Lund University, Skåne University Hospital, Lund, Sweden; 2 Department of Medicine, Karolinska Institutet, Karolinska University Hospital, Stockholm, Sweden; 3 Department of Cardiology, Lund University, Skåne University Hospital, Lund, Sweden

## Background

The ultimate goal of acute reperfusion therapy in patients suffering from acute coronary occlusion is to accomplish as much myocardial salvage as possible. In order to determine the myocardial salvage index, the extent of infarction needs to be related to the myocardium at risk (MaR). Thus, the ability to assess both infarct size and MaR is of central clinical and scientific importance, especially when designing clinical trials aimed at evaluating the cardioprotective efficiency of different acute interventions. The aim of the present study was to explore the relationship between T2-weighted cardiac magnetic resonance (CMR) and early gadolinium enhanced steady state free precession (EGE) CMR for determination of MaR in patients with acute myocardial infarction.

## Methods

Twenty-one prospectively included patients with first-time ST-elevation myocardial infarction underwent CMR 1 week after primary percutaneous coronary intervention. T2-weighted images, for assessment of MaR, were acquired before injection of a gadolinium-based contrast agent. After contrast injection, EGE images were acquired for assessment of MaR and late gadolinium enhancement images were acquired for assessment of infarct size.

## Results

Myocardium at risk by T2-weighted imaging and EGE was 29 ± 11% and 32 ± 12% of the left ventricle, respectively. Thus, MaR with T2-weighted imaging was slightly smaller than MaR by EGE (-3.0 ± 3.9%; p < 0.01). There was a significant correlation between the two MaR measures (r2 = 0.89, p < 0.01). Furthermore, no significant difference in the myocardial salvage index, calculated using T2-weighted imaging and EGE for MaR (56 ± 22% vs 58 ± 23%, p = 0.18), was found.

## Conclusions

There is a strong agreement between MaR assessed by T2-weighted imaging and MaR assessed by EGE in patients with reperfused acute myocardial infarction 1 week after the acute event. Thus, both methods can be used to determine MaR and myocardial salvage at this point in time.

## Funding

This study has been funded by the Swedish Research Council, the Swedish Heart and Lung Foundation, the Medical Faculty at Lund University, Sweden, European Commission FP7 Consortium CardioCell, Region of Scania, Sweden, the Stockholm County Council and Karolinska Institutet/Stockholm County Council Strategic Cardiovascular Program.

